# A new meta-heuristic pathfinder algorithm for solving optimal allocation of solar photovoltaic system in multi-lateral distribution system for improving resilience

**DOI:** 10.1007/s42452-020-04044-8

**Published:** 2021-01-12

**Authors:** Varaprasad Janamala

**Affiliations:** Department of Electrical and Electronics Engineering, School of Engineering and Technology, CHRIST (Deemed to be University), Bengaluru, Karnataka 560 074 India

**Keywords:** Distributed generation, Interline-photovoltaic system, Power loss minimization, Radial distribution system, Pathfinder algorithm, Meta-heuristic algorithms

## Abstract

A new meta-heuristic Pathfinder Algorithm (PFA) is adopted in this paper for optimal allocation and simultaneous integration of a solar photovoltaic system among multi-laterals, called interline-photovoltaic (I-PV) system. At first, the performance of PFA is evaluated by solving the optimal allocation of distribution generation problem in IEEE 33- and 69-bus systems for loss minimization. The obtained results show that the performance of proposed PFA is superior to PSO, TLBO, CSA, and GOA and other approaches cited in literature. The comparison of different performance measures of 50 independent trail runs predominantly shows the effectiveness of PFA and its efficiency for global optima. Subsequently, PFA is implemented for determining the optimal I-PV configuration considering the resilience without compromising the various operational and radiality constraints. Different case studies are simulated and the impact of the I-PV system is analyzed in terms of voltage profile and voltage stability. The proposed optimal I-PV configuration resulted in loss reduction of 77.87% and 98.33% in IEEE 33- and 69-bus systems, respectively. Further, the reduced average voltage deviation index and increased voltage stability index result in an improved voltage profile and enhanced voltage stability margin in radial distribution systems and its suitability for practical applications.

## Introduction

Electrical distribution system (EDS) normally characterized by higher R/X ratio and radiality, as well as operation at low voltage profile with high currents. Most of the distribution systems serve inductive loads which results in low voltage profile and consequently higher distribution losses. Increased power loss implies inefficient operation not only from a technical point of view but also from economical aspects due to more power generation requirement and consequently its associated cost. Particularly in India, the transmission and distribution losses are around 20% of the total power generation [1]. In order to overcome this potential problem, integration of distribution generation (DG) in EDS becomes inevitable, which can lead to improvement of the performance, power quality, stability and reliability [2]. Also, the concept of DG can contribute to increase in the usage of renewable energy (RE) such as photovoltaic (PV) and wind turbine (WT) sources by which greenhouse gas (GHG) emission can be minimized in power system operation. From the definitions, DG sizes can vary from 1 kW to 5 MW and are suitable for integration even at small consumer sites. This may contribute to avoid investments in construction of new larger generation plants and transmission lines. Integration of DG units at appropriate locations and their capacities is a complex non-convex optimization problem with multiple-objectives and has been addressed exhaustively using various conventional (non-heuristic) and heuristic search algorithms (HSAs) [3–5]. However, in view of complexity and computational time, HSAs have become more popular than conventional approaches and also have been identified for many engineering optimization problems. HSAs are simple to understand, easy to implement with no need of any derivations and require only a few parameters as inputs (like search space dimension, variable limits and maximum number of iterations) without modifications in their basic structure. In [6], different operators of evolutionary algorithms (EA) are adopted to form the hybrid grey wolf optimizer (HGWO) and applied to solve the OADG problem for loss minimization. The case studies are performed on IEEE 33-, 69- and Indian 85-bus practical systems for finding the optimal location and sizes of different types of DG technologies. In [7], by merging another local search algorithm, an enhanced genetic algorithm (EGA) is proposed for solving simultaneous allocation of DGs and capacitors for loss minimization. The simulations are performed on IEEE 33-, 69-, and 119-bus test distribution networks by constraining DG capacity to 50% of total load and 100% reactive power compensation via capacitors. In [8], weight improved particle swarm optimization algorithm (WIPSO) and self adaptive differential evolution algorithm (SADE) are proposed along with distributed generation sitting index (DGSI) ranking method towards loss minimization via simultaneous allocation of DGs and capacitors. In [9], whale optimization algorithm (WOA) is proposed for optimal allocation of different kinds of single DG unit radial distribution system. The search space for WOA is reduced using power loss index (PLI) and DG size is optimized for loss minimization by considering a specified range of DG size. In [10], real power loss indices are used to limit the search space of location of DGs and techno-economic aspects are optimized in distribution system operation using shuffled frog leaping algorithm (SFLA). In [11], multi-objective whale optimization algorithm (MOWOA) and analytical hierarchy process (AHP) is proposed for solving multi-objective optimization problem formulated for renewable DG allocation in multi-type consumers connected distribution system towards minimizing the real power loss index, voltage stability index and cost benefits. In [12], the elitism phase of ant lion optimization (ALO) is updated using PSO, and fuzzy logic controller (FLC) is proposed to minimize the error criterion. The hybrid approach is used to find optimal rating and location of renewable DGs in IEEE 33-bus system for minimizing loss, operating cost, voltage deviation and inverse of voltage stability index. In [13], ant lion optimization (ALO) is proposed for optimal allocation of different kinds of single DG unit radial distribution system. The search space for WOA is reduced using index vector method (IVM). In [14], gbest-guided artificial bee colony (GABC) along with index vector method (IVM) and power loss index (PLI) methods are proposed for multi-objective optimization problem of DGs integration in IEEE 33- and 85-bus systems. Whale optimization algorithm (WOA) is proposed for integrating DGs considering multi-objectives and simulations performed on IEEE 33-bus and 69-bus systems [15]. In [16], loss sensitivity factors (LSF) and normalized voltage profile are used to predefine candidate locations for DGs integration. Later, dragonfly algorithm (DA) is implemented to determine optimal location and size of single DG considering loss minimization as the major objective. In [17], bat algorithm (BA) is proposed for integrating solar PV type DG towards loss minimization. A predefined size of PV array is taken as the control variable and optimized for a number of arrays. In [18], a hybrid approach is proposed using weight improved PSO (WIPSO) and gravitational search algorithm (GSA) called hybrid WIPSO-GSA for techno-economic benefits in terms of minimum total cost, voltage stability and maximum loadability with DGs in IEEE 33- and 85-bus systems. In [19], LSFs based potential locations are ranked for DG and capacitors integration, and later their optimal locations and sizes are determined by applying moth–flame optimization (MFO) for reducing the loss-voltage-cost index (LVCI). In [20], a multi-objective function using loss, voltage deviation and voltage stability index is formulated for renewable DGs allocation. The locations are prioritized using LSFs and later ALO is applied for deducing best locations and sizes. In [21], a hybrid approach using grasshopper optimization and cuckoo search (GOA-CSA) is proposed for solving optimal allocation of DGs considering real power loss, voltage deviation and real and reactive power cost of DGs, in which the sizes of DGs are constrained by specific limits. In [22], hybrid harmony search algorithm (HSA) and particle artificial bee colony algorithm (PABC) HSA–PABC is proposed for integrating DGs optimally in the network. The search space for locations is determined using LSI and optimized for minimum real power loss. Also, the impact of DGs is evaluated using voltage deviation (VDI) and voltage stability index (VSI). In [23], fixed sizes of PV, WT and capacitors are chosen and their optimal number along with a single biomass DG location and sizes are optimized using multi-objective PSO (MOPSO) considering loss, voltage stability and voltage deviation. Later, FLC is used as a trade-off solution set. The simulations are performed considering different load profiles w.r.t weather seasons and corresponding DG output power variation, losses and voltage profiles are analyzed. In [24], Symbiotic Organisms Search (SOS) algorithm is proposed for reducing the energy losses w.r.t seasons by installing renewable DGs. The technical and economic benefits are compared with other HSAs namely GA, PSO and firefly algorithm (FFA). In [25], based on technical and economical indexes, an analytic hierarchy process (AHP) is proposed for determining the weighting factors for multi-objectives. The predefined search space for DG locations is determined using a combined sensitivity index, formed with apparent load power and voltage deviation. Further, the DGs are allocated under different loading levels using PSO. Similarly, spring search algorithm (SSA) [26] and water cycle algorithm (WCA) [27] are proposed for simultaneous allocation of renewable DGs and capacitors considering the techno-economic multi-objective function.

On the other side, network reconfiguration (NR) is another promising approach for obtaining smooth load profiles across all sections in the network, avoiding faulty sections and consequently improving the resilience in distribution system operation and control. Apart from technical benefits, optimal network reconfiguration (ONR) approach has also some disadvantages from a practical perspective. It needs to equip mechanical switches for every branch and infrastructure for automatic control. Also, ONR needs high investment cost and regular maintenance for mechanical switches, which may not be feasible practically for all EDSs. However, many researchers have analyzed the impact of simultaneous OADG and optimal network reconfiguration (ONR) considering technical aspects [28]. In [29], harmony search (HS) and teaching–learning-based optimization (TLBO) are hybridized for forming comprehensive teaching learning harmony search optimization algorithm (CTLHSO) and applied to solve simultaneous DGs and reconfiguration problem for minimizing the loss and voltage deviation from reference bus considering different loading levels. In [30], improved sin-cosine algorithm (SCA) with levy flights is proposed and optimized for multi-objective function with loss and voltage stability index via determining the optimal branches to open and tie-lines to close, for forming optimal reconfiguration and location and sizes of DGs. In [31], a new thief and police algorithm (TPA) is proposed and applied for solving the renewable DGs and capacitor allocation along with the reconfiguration problem considering loss, voltage stability and operational cost. In [32], three different HSAs namely integrated PSO (IPSO), TLBO and Jaya optimization enhance the voltage stability and minimize the real power loss in distribution system operation via simultaneous optimal allocation of DGs and reconfiguration. In [33], TLBO is proposed for solving the OADG problem considering techno-economic objectives. In [34], an improved Elitist–Jaya (IEJAYA) algorithm is proposed for minimizing the real power losses and loadability enhancement via joint optimal location and rating of DGs and reconfiguration of distribution system. In [35], Salp Swarm Algorithm (SSA) is implemented for minimizing the losses and voltage deviation in solving the simultaneous OADG and ONR problem together in the distribution system.

At this stage, it is worthwhile to realize the following challenges in the OADG problem. Location and sizes of DGs are the main control variables in the search space. In most of the works, the DG sizes are constrained by a capacity limit [7–9, 11–13, 15, 22–24, 26] and locations are limited for predefined candidate buses using different sensitivity indices [8, 10, 14, 16–19] and randomly selected [18]. In some other works, the DG sizes are unconstrained (but the sum of their total capacities should not be more than total system demand), and all buses in the network are considered as search space for locations [6, 12, 19–21]. On the other hand, identification of appropriate branches for opening and tie-lines for closing is additional search space in simultaneous OADG and ONR problem. In comparison, the first type of works may seem to be efficient w.r.t. convergence time by having limited search space, whereas, the second type of works can result in global optima (w.r.t. high utilization of renewable DG power in distribution systems and large search space), by which the efficiency and redundancy characteristics of a HSA can be evaluated and compared comprehensively.

Notably, OADG and/or ONR may not be of ensuring maximum utilization of RE based DGs and lead to curtailment due to various operational constraints. In order to improve resilience and reduce RE curtailment rate in operation and control of EDS, integration of RE to multiple feeders/laterals is essential, which has not been paid much attention to in the literature. Interline-Photovoltaic (I-PV) concept [36] and soft open points (SOPs) [37] are some of such approaches, by which it is possible to maximize utilization of RE among different feeders/locations and ensure resilience in operation and control.

On the other side, there is no single specific algorithm which can solve all types of optimization problems as proved in the no-free-launch theorem [38]. Hence, the researchers are still aspiring to introduce new heuristic algorithms and also improvements to the existing algorithms for solving different kinds of optimization problems. In this paper, the effectiveness of a new meta-heuristic optimization algorithm, namely Pathfinder Algorithm (PFA) [39] is proposed for solving the OADG problem and compared with the unconstrained search space for location and sizes of DGs. The main advantage of PFA is that it needed only two variables (i.e., population and the number of iterations) as controlling parameters and was easy to implement. Later, optimal I-PV configuration for addressing the need of interoperability of DGs among multi-laterals is proposed using the proposed PFA. As per this authors’ knowledge, this work is the first kind of application for PFA in solving the simultaneous OADG and I-PV problem and can claim as a main contribution in this research area. Considering loss minimization, the maximum possible penetration levels of solar PV type DG are determined without compromising in technical as well as radiality constraints of the distribution network. In [39], the application of PFA for solving optimal allocation of RE sources in IEEE 30-bus power system is presented by considering multi-objectives OPF framework and proves its superiority over other existing solutions like ABC, Fuzzy-PSO, N-R and Fuzzy-GA. But, the effectiveness of the proposed PFA is still needed to examine the optimization problems with continuous and discrete variables simultaneously and this research work is one such attempt.

The rest of the paper is organized as follows. Section 2 explains the concept of interline-photovoltaic (I-PV) system and its mathematical modeling. Section 3 describes the problem statement, objective function and its equal and unequal constraints. Section 4 presents a brief review of major stages involved in the Pathfinder Algorithm (PFA) and its mathematical formulations. Also it covers the overall procedure applied for solving the DG allocation problem using PFA. Section 5 explains the effectiveness of PFA in solving optimal I-PV configuration in IEEE 33- and 69-bus systems, and Sect. 6 concludes the paper with major contributions and research findings by the proposed methodology.

## Proposed interline photovoltaic system

**Fig. 1:**
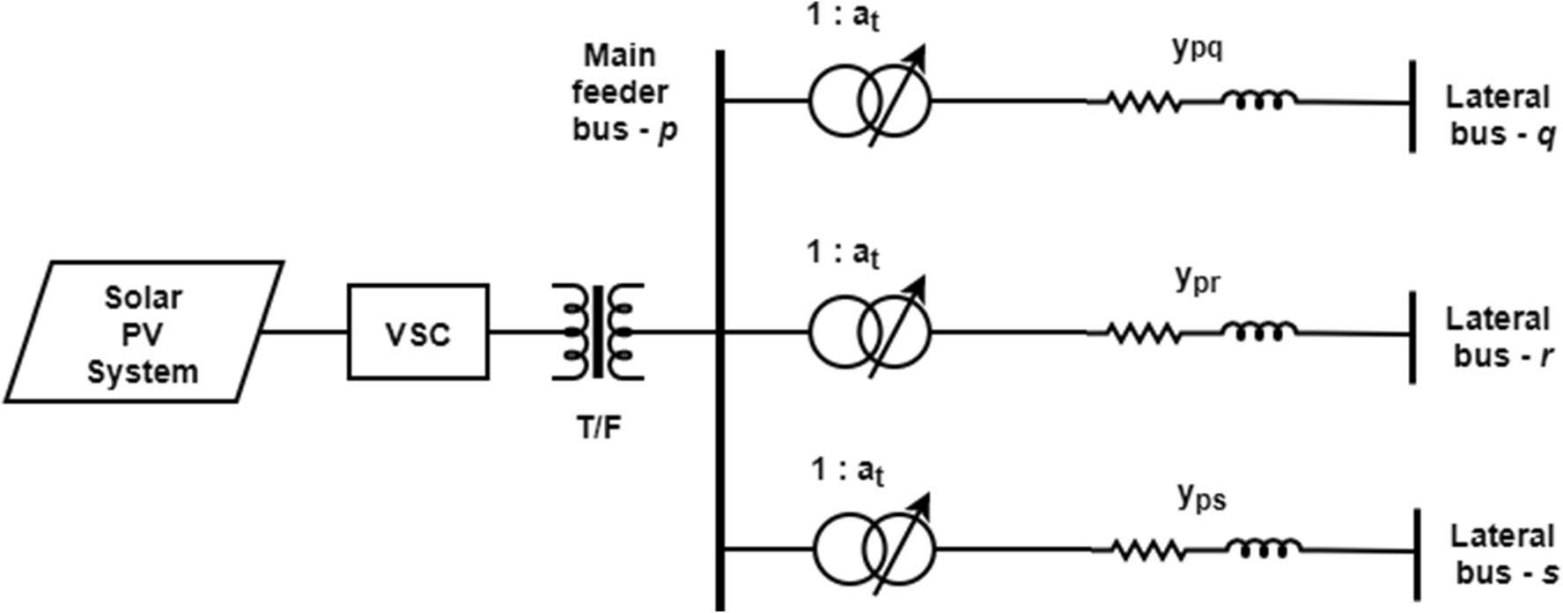
Schematic diagram of the proposed I-PV system

Based on the meteorological conditions, some locations are suitable for installing a specific type of RE units having a large scale capacity. But the high penetrations levels of RE at a single location can cause degradation of the feeder performance considerably. In order to avoid this problem, the Interline–Photovoltaic (I–PV) concept is highly adaptable to inject the total yielded PV power at multiple points into the network and consequently for power flow control and management between two adjacent feeders. As introduced in [36], the I-PV system consists of a common PV source for the different Voltage Source Converters (VSC) which are used to inject PV output power into the AC grid via shunt coupling transformers (T_sh_). In this work, the configuration of basic I-PV system is modified for easy adoption in the conventional load flow studies as shown in Fig. 1.

Under steady-state operating conditions, the I-PV system can be assumed as P + type DG, which is connected to the main feeder AC bus *p*. This common bus is interconnected to various lateral feeders via tap-changers (T_sh_) and a distribution line having an impedance of Z. The real and reactive power injections of the I-PV system at different buses on lateral feeders can be regulated optimally by setting the tap-ratios.

**Fig. 2:**
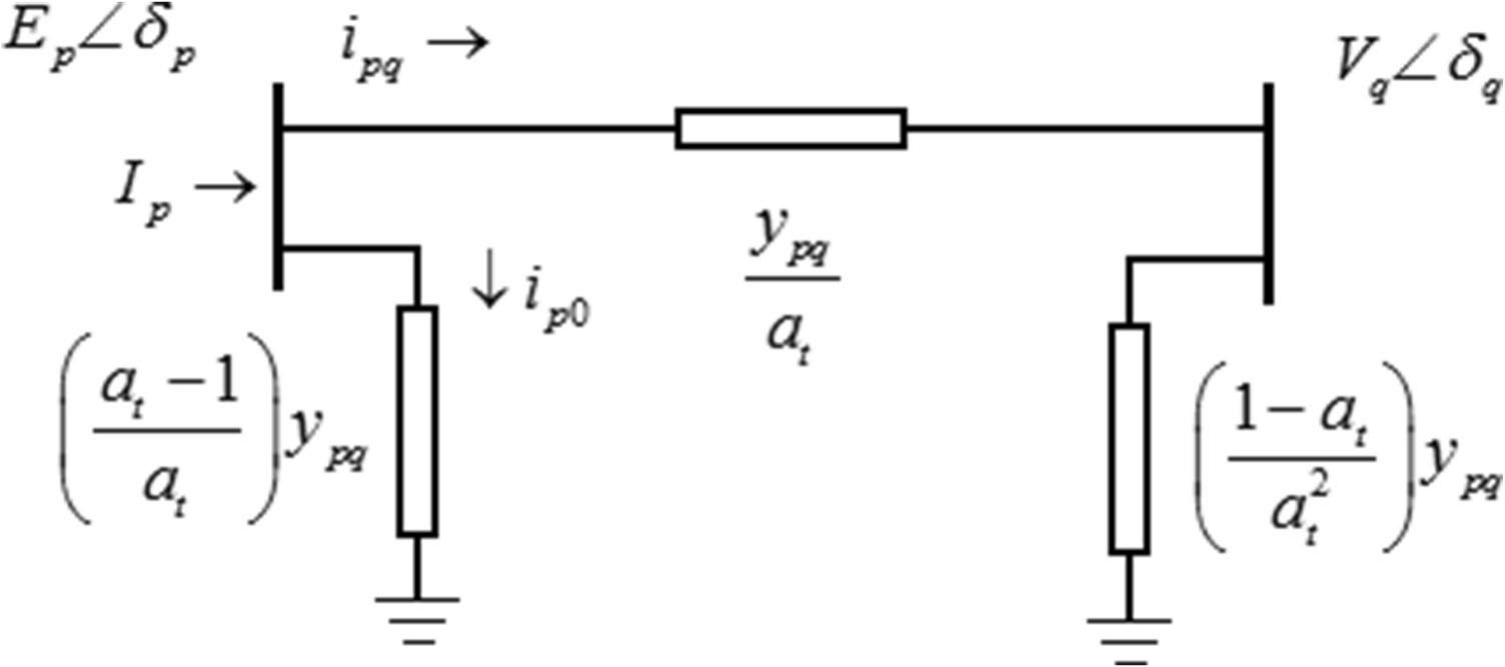
Equivalent circuit of a branch between main feeder bus-*p* and lateral bus-*q*

The branch admittance between main feeder bus-*p* and lateral bus-*q* is modeled in π-model, as given in Fig. 2.

The current at bus-*p*, and defined positive in the direction *p → q* is given by,

1 $$I_{p} = i_{pq} + i_{p0} = \left( {E_{p} \angle \delta_{p} - V_{q} \angle \delta_{q} } \right)\frac{{\left( {y_{pq} \angle \theta_{pq} } \right)}}{{a_{t} }} + E_{p} \angle \delta_{p} \left( {\frac{{a_{t} - 1}}{{a_{t} }}} \right)\left( {y_{pq} \angle \theta_{pq} } \right)$$

where $I_{p}$ is the current drawn at bus-*p* through interconnection between bus-*p* and bus-*q*, $E_{p}$ and $V_{q}$ are the voltage magnitudes at bus-*p* and bus-*q* respectively, $\delta_{p}$ and $\delta_{q}$ are the load angles at bus-*p* and bus-*q* respectively, $y_{pq}$ and $\theta_{pq}$ admittance and its angle of branch between bus-*p* and bus-*q* respectively, $a_{t}$ ratio of tap-changer in series with the branch connected between bus-*p* and bus-*q*.

The complex power $S_{pq}$, extracted at bus-*p* is through branch p *→ q* is given by,

2 $$S_{pq} = E_{p} \angle \delta_{p} \left( {I_{p} } \right)^{*}$$

3 $$S_{pq} = \left| {E_{p} } \right|^{2} \left| {y_{pq} } \right|\angle - \theta_{pq} - \left\{ {\left| {E_{p} } \right|\left| {V_{q} } \right|\frac{{\left| {y_{pq} } \right|}}{{a_{t} }}\angle \left( {\delta_{pq} - \theta_{pq} } \right)} \right\}$$

The real power and reactive powers supplied by the I-PV system can be determined by adding power extractions through all branches connected from main feeder bus-p to lateral feeders and are given by,

4 $$P_{I - PV} = \sum\limits_{{k\left( {pq} \right) = 1}}^{{n_{l} }} {P_{{k\left( {pq} \right)}} } = \sum\limits_{{k\left( {pq} \right) = 1}}^{{n_{l} }} {\left\{ {\left| {E_{p} } \right|^{2} \left| {y_{pq} } \right|\cos \left( {\theta_{pq} } \right) - \left| {E_{p} } \right|\left| {V_{q} } \right|\frac{{\left| {y_{pq} } \right|}}{{a_{t} }}\cos \left( {\delta_{pq} - \theta_{pq} } \right)} \right\}}$$

5 $$Q_{I - PV} = \sum\limits_{{k\left( {pq} \right) = 1}}^{{n_{l} }} {Q_{{k\left( {pq} \right)}} } = \sum\limits_{{k\left( {pq} \right) = 1}}^{{n_{l} }} {\left\{ { - \left| {E_{p} } \right|^{2} \left| {y_{pq} } \right|\sin \left( {\theta_{pq} } \right) - \left| {E_{p} } \right|\left| {V_{q} } \right|\frac{{\left| {y_{pq} } \right|}}{{a_{t} }}\sin \left( {\delta_{pq} - \theta_{pq} } \right)} \right\}}$$

where $P_{{I{ - }PV}}$ and $Q_{{I{ - }PV}}$ are the real and reactive power supplied by the I-PV system respectively, $n_{l}$ is the number of braches connected with lateral feeders from PV bus, and $k\left( {pq} \right)$ is the branch index connected between bus-*p* and bus-*q*.

And subsequently, the MVA rating and operating power factor of VSC of the I-PV system are given by,

6 $$S_{{I{ - }PV}} = \sqrt {P_{{I{ - }PV}}^{2} + Q_{{I{ - }PV}}^{2} }$$

7 $$pf_{{I{ - }PV}} = {{P_{{I{ - }PV}} } \mathord{\left/ {\vphantom {{P_{{I{ - }PV}} } {S_{{I{ - }PV}} }}} \right. \kern-0pt} {S_{{I{ - }PV}} }}$$

In conventional NR load flow studies, the selected bus-p on main feeder for integrating a solar PV system is converted as generator bus i.e., PV bus. The real power generation ($P_{{I{ - }PV}}$), its voltage magnitude ($\left| {E_{p} } \right|$) and ratios of tap-changers ($a_{t}$) are considered as control variables in optimization problem.

## Problem formulation

In this paper, the location/bus and power injection by SPV system and locations on lateral feeders to form I-PV configuration are the main control variables. The impact of the I-PV system on the performance of RDS is evaluated in terms of real power loss, voltage profile and voltage stability.

### Objective function

Loss minimization in RDS is an important operational requirement for improving the utilization of DG’s power or reducing grid-dependency. The total real power loss in a distribution system is given by,

8 $$OF = \hbox{min} f\left( {P_{loss} } \right) = \sum\limits_{{k_{{\left( {mn} \right)}} = 1}}^{{n_{br} }} {r_{\left( k \right)} \left( {\frac{{P_{\left( n \right)}^{2} + Q_{\left( n \right)}^{2} }}{{\left| {V_{\left( n \right)} } \right|^{2} }}} \right)}$$

Also, operating the distribution system at a proper voltage profile and consequently adequate voltage stability margin is another essential requirement for having quality and reliability supply.

### Operational constraints

The following constraints are considered in solving the proposed objective function.

- Bus voltage constraint: The voltage magnitude of each bus should be maintained within specified limits,
  9 $$\left| {V_{\left( n \right)} } \right|_{\hbox{min} } \le \left| {V_{\left( n \right)} } \right| \le \left| {V_{\left( n \right)} } \right|_{\hbox{max} } \quad n = 1,2, \ldots ,n_{b}$$
- Thermal constraint: The current flow through any branch should not be more than its maximum rated limit,
  10 $$\left| {I_{\left( k \right)} } \right| \le \left| {I_{\left( k \right)} } \right|_{\hbox{max} } \quad k = 1,2, \ldots ,n_{br}$$
- I-PV active power compensation constraint: The total real power generation via DG in the network should not be more than total real power demand of the system,
  11 $$P_{I - PV} \le P_{load\left( T \right)}$$
- I-PV reactive power compensation constraint: The total reactive power generation via WTs or capacitors in the network should not be more than total reactive power demand of the system,
  12 $$Q_{I - PV} \le Q_{load\left( T \right)}$$
- I-PV bus voltage constraint: The reactive power generation via VSC of I-PV system can be controlled by regulating the bus-p voltage within specified limits.
  13 $$\left| {E_{p} } \right|_{\hbox{min} } \le \left| {E_{p} } \right| \le \left| {E_{p} } \right|_{\hbox{max} }$$
- Tap-ratio constraint: The ratios of tap-changers associated with different interconnections between I-PV bus and lateral buses are constrained by,
  14 $$a_{t,k}^{\hbox{min} } \le a_{t,k} \le a_{t,k}^{\hbox{max} } ,\quad k = 1,2, \ldots ,n_{l}$$
- Radiality constraint: The number of branches and their interconnections in a radial distribution network should not create loops, and is considered as,
  15 $$n_{br} = n_{b} - 1$$
  where $P_{loss}$ is the total real power loss in the feeder distribution, *k* is the index of a branch connected between buses $m$ and $n$; $P_{\left( n \right)}$, $Q_{\left( n \right)}$, $\left| {V_{\left( n \right)} } \right|$ are the real, reactive power loads and voltage magnitude of $n$ th bus; $n_{br}$ and $n_{b}$ are the number of branches and buses of the network respectively; $\left| {I_{\left( k \right)} } \right|$ is the branch current; $P_{I - PV}$ and $Q_{I - PV}$ are the active and reactive power injections by I-PV system respectively; $P_{load\left( T \right)}$ and $Q_{load\left( T \right)}$ are the active and reactive power loading on the network respectively.

### Average voltage deviation index

The impact of optimal I-PV system configuration on network voltage profile is determined using average voltage deviation index (AVDI) w.r.t. substation voltage and defined mathematically as,

16 $$AVDI = \frac{1}{{n_{b} }}\left\{ {\left[ {\sum\limits_{q = 1}^{{n_{b} }} {\sqrt {\left( {V_{{\left( {ref} \right)}} - \left| {V_{(q)} } \right|} \right)^{2} } } } \right]} \right\}.$$

### Voltage stability analysis

For maintaining secured and reliable operation, assessment voltage stability is very important. In this paper, the voltage stability index (VSI) proposed in [40] is adopted and determined to identify the closeness of the distribution system to voltage collapse. Mathematically, VSI is defined as,

17 $$VSI_{\left( q \right)} = \left| {V_{(q)} } \right|^{4} - 4\left( {x_{{\left( {pq} \right)}} P_{\left( q \right)} - r_{{\left( {pq} \right)}} Q_{\left( q \right)} } \right)^{2} - 4\left( {r_{{\left( {pq} \right)}} P_{\left( q \right)} + x_{{\left( {pq} \right)}} Q_{\left( q \right)} } \right)\left| {V_{(p)} } \right|^{2} \ge 0$$

For stable operation, the VSI of a load bus should be more than zero and the lowest value among all buses is treated as system voltage stability index.

In addition to the reduced real power loss, the impact of proposed I-PV configuration on the distribution system can be understood more clearly by observing AVDI and VSI values. The reduced $AVDI$ and increased $VSI$ can indicate improved voltage profile across the network and enhanced voltage stability margin.

## Pathfinder algorithm

In nature, some groups of animals often migrate to different locations as per the seasons by following social hierarchy amongst them. Foraging, exploiting and hunting behaviors of a group of animals are the major motivational factors in developing the Pathfinder Algorithm (PFA) [39]. Also, playing a lead role in a swarm for successful hunting and consequently influencing the other individuals to follow it, are the features of the computational process of PFA. The proposed PFA saves the best position achieved so far as the position of pathfinder and it never gets lost. The pathfinder is skilled to explore and exploit the hunt or food source. Different individuals follow the pathfinder and collaborate with their neighbor, so they can explore and exploit the objective in search space. The controlling parameters can keep the PFA from the possibility of local optima. Hence, PFA can be solved optimization problems effectively. In this section, the mathematical model involved for initialization, iteration and stopping phases of PFA are covered.

### Mathematical model of PFA

In an $n$-dimensional search space, an individual animal from a swarm equal to the number of search variables is located in a best hunting area for a prey in a time is treated as leader and named as pathfinder. This stage is similar to finding the initial best fitness value among all solutions obtained using the initial population at the initialization stage of any HSA. The initial population is generated using Eq. (18), in which $x_{i\left( 0 \right)}$ is position vector of individual animal $i$ at initial stage, *d* is the dimension of search space, *L*_*b*_ and *U*_*b*_ are the lower and upper boundaries of the variables in the optimization problem.

18 $$x_{i\left( 0 \right)} \left( t \right) = L_{b} + \left( {U_{b} - L_{b} } \right) \cdot *rand\left( {1, d} \right)$$

Now the behavior of all other followers w.r.t change in their position and time is modeled as given in Eq. (19).

19 $$x_{i\left( k \right)} \left( {t + \Delta t} \right) = x_{i\left( 0 \right)} \left( t \right) \cdot \vec{a} + f_{ij} + f_{p} + \nu_{v}$$

where $t$ is time, $x_{i\left( 0 \right)}$ and $x_{i\left( k \right)}$ is the position vector of individual animals $i$ at initial stage and at iteration $k$ respectively; the $\vec{a}$ is the unit vector of zero angle, $f_{ij}$ is the interaction between a pair of neighbors $i$ and $j$; $f_{p}$ is the global best so far or pathfinder fitness; and $\nu_{v}$ is the vibration vector.

Simultaneously the position pathfinder is updated by using Eq. (20).

20 $$x_{p\left( k \right)} \left( {t + \Delta t} \right) = x_{p\left( 0 \right)} \left( t \right) + \Delta x_{P} + \nu_{f}$$

$x_{p\left( 0 \right)}$ and $x_{p\left( k \right)}$ is position vector of pathfinder $p$ at initial stage and at iteration $k$ respectively; $\Delta x_{P}$ is position change by pathfinder and $\nu_{f}$ is vector of fluctuation rate.

By modifying Eqs. (19) and (20) to Eqs. (21) and (22) for solving an optimization problem, the following equations are proposed for collective movement of swam.

21 $$x_{i} \left( {k + 1} \right) = x_{i} \left( k \right) + \alpha r_{1} \cdot \left[ {x_{j} \left( k \right) - x_{i} \left( k \right)} \right] + \beta r_{2} \cdot \left[ {x_{p} \left( k \right) - x_{i} \left( k \right)} \right] + \nu_{v} ,\quad i \ge 2$$

22 $$x_{p} \left( {k + 1} \right) = x_{p} \left( k \right) + 2r_{3} \cdot \left[ {x_{p} \left( k \right) - x_{p} \left( {k - 1} \right)} \right] + \nu_{f}$$

23 $$\nu_{v} = \left( {1 - \frac{k}{{k_{\hbox{max} } }}} \right) \cdot u_{1} \cdot D_{ij} ,\,\,D_{ij} = \left\| {x_{i} - x_{j} } \right\|\quad {\text{and}}\quad \nu_{f} = u_{2} \cdot e^{{\left( {\frac{ - 2k}{{k_{\hbox{max} } }}} \right)}}$$

where $r_{1}$, $r_{2}$ and $r_{3}$ are uniformly distributed random numbers in [0, 1]; $u_{1}$ and $u_{2}$ are the random vectors in the range of [− 1, 1]; $\alpha$ is the interaction coefficient for defining the magnitude of interaction with a neighbor, $\beta$ is the attraction coefficient for setting the random distance for an individual with group, preferably with pathfinder, $k_{\hbox{max} }$ is maximum number of iteration. The range of $\alpha$ and $\beta$ is [1, 2]. In specific, $\nu_{v}$ and $\nu_{f}$ are generated in each iteration for random walk in multi-dimension for each animal in the group. The more understanding on swam movements w.r.t. changes of $\alpha$,$\beta$, $u_{1}$ and $u_{2}$ in PFA is illustrated in [34].

### Implementation procedure of PFA for solving optimal I-PV configuration

The solution methodology of PFA for solving the optimal I-PV configuration considering resilience and radiality constraints is followed in the following sequential steps.

#### Initialization of animal swarm

In the PFA, a population of animal swarm is represented by $X = \left[ {x_{1} ,x_{2} , \ldots ,x_{d} } \right]^{T}$, where $x_{i}$, $i \in \left\{ {1,2, \ldots ,d} \right\}$ represents a possible solution vector and it is consider in optimal I-PV configuration problem as follows:

24 $$x_{i} = \left[ {l_{PV,1} ,l_{PV,2} , \ldots ,l_{{PV,n_{PV} }} ,P_{PV,1} ,P_{PV,2} , \ldots ,P_{{PV,n_{PV} }} ,l_{IPV,1} ,l_{IPV,2} , \ldots ,l_{{IPV,n_{IPV} }} ,o_{br,1} ,o_{br,2} , \ldots ,o_{{br,n_{IPV} }} } \right]$$

where $l_{PV,i}$, $P_{PV,i}$, $l_{IPV,i}$ and $o_{br,i}$, respectively, represent the locations for PV system on main feeder, the size of PV system,, the locations on lateral feeders for interconnection and the branches for opening towards radiality constraint. $n_{PV}$ and $n_{IPV}$, respectively, the number of PV locations on main feeder and the number of buses on lateral feeders for I-PV configuration. In this paper, $n_{PV}$ is fixed and it is 1 and whereas $n_{IPV}$ is chosen differently in different case studies.

In the PFA, the animal swarm initiates randomly within specified lower and upper bounds and thus the possible solution variables are generated as follows:

25 $$l_{PV,i} = round\left[ {l_{PV,\hbox{min} } + \left( {l_{PV,\hbox{max} } - l_{PV,\hbox{min} } } \right) \cdot *rand\left( {1, n_{PV} } \right)} \right]\quad i \in \left\{ {1,2, \ldots ,n_{PV} } \right\}$$

26 $$l_{IPV,i} = round\left[ {l_{IPV,\hbox{min} } + \left( {l_{IPV,\hbox{max} } - l_{IPV,\hbox{min} } } \right) \cdot *rand\left( {1, n_{IPV} } \right)} \right]\quad i \in \left\{ {1,2, \ldots ,n_{IPV} } \right\}$$

27 $$O_{br,i} = round\left[ {o_{br,\hbox{min} } + \left( {o_{br,\hbox{max} } - o_{br,\hbox{min} } } \right) \cdot *rand\left( {1, n_{IPV} } \right)} \right]\quad i \in \left\{ {1,2, \ldots ,n_{IPV} } \right\}$$

28 $$P_{PV,i} = \left[ {P_{PV,\hbox{min} } + \left( {P_{PV,\hbox{max} } - P_{PV,\hbox{min} } } \right) \cdot *rand\left( {1, n_{PV} } \right)} \right]\quad i \in \left\{ {1,2, \ldots ,n_{PV} } \right\}$$

Here the continuous variables for locations (i.e., $l_{PV,i}$ and $l_{IPV,i}$) and branch numbers (i.e., $o_{br,i}$) are rounded for discrete variables as observed in Eqs. (25)–(27) and used in the moving functions.

#### Cost function

In the PFA, the randomly generated positions of each animal are evaluated by a cost function as defined in Eq. (29).

29 $$CF_{T} = OF + k_{v} \sum\limits_{i = 1}^{{n_{b} }} {\left( {\left| {V_{{\left( {\lim } \right)}} } \right| - \left| {V_{\left( i \right)} } \right|} \right)^{2} + k_{i} \sum\limits_{i = 1}^{{n_{br} }} {\left( {\left| {I_{{\left( {\lim } \right)}} } \right| - \left| {I_{\left( i \right)} } \right|} \right)^{2} } }$$

where $k_{v}$ and $k_{i}$ are penalty factors for maintaining bus voltage magnitudes, $\left| {V_{\left( i \right)} } \right|$ and branch currents, $\left| {I_{\left( i \right)} } \right|$ within specified limits as given in Eqs. (9) and (10), respectively.

In each iteration, the solution vector generated by the PFA can change the values of bus voltage magnitudes and branch currents and subsequently the cost function evaluates by adjusting dependent variable to its violated limit as given by general way in Eq. (30).

30 $$v_{\lim } = \left\{ {\begin{array}{*{20}l} {v_{\hbox{max} } } \hfill & {\quad \forall \left( {v > v_{\hbox{max} } } \right)} \hfill \\ {v_{\hbox{min} } } \hfill & {\quad \forall \left( {v < v_{\hbox{min} } } \right)} \hfill \\ v \hfill & {\quad \forall \left( {v_{\hbox{min} } \le v \le v_{\hbox{max} } } \right)} \hfill \\ \end{array} } \right.$$

**Fig. 3:**
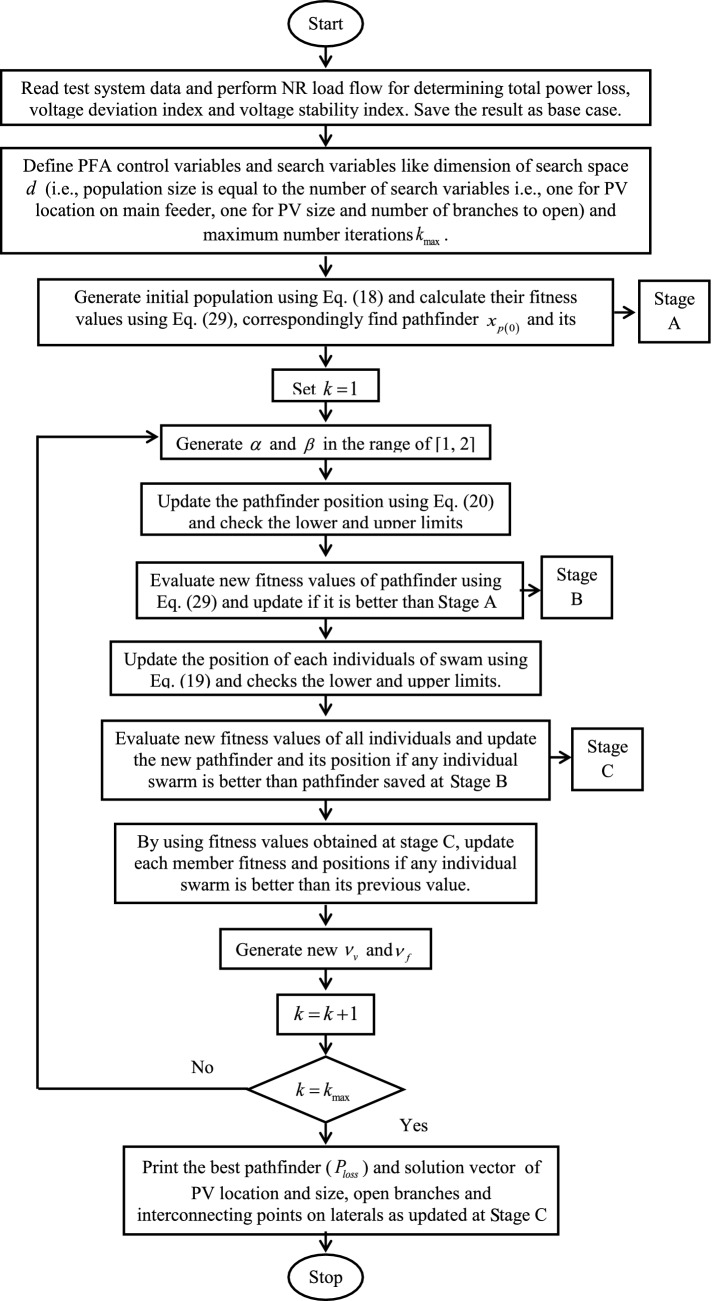
Flowchart of proposed PFA for solving optima I-PV configuration

The complete solution methodology of PFA for obtaining the optimal I-PV configuration considering radiality constraint for improving resilience is given as a flowchart in Fig. 3.

## Results and discussion

The proposed methodology is applied on IEEE 33-bus [41] and IEEE 69-bus systems [42]. The simulations are performed for three different scenarios: (1) optimal location and sizing of PV system, (2) optimal I-PV configuration with radiality constraint, and (3) optimal I-PV configuration without radiality constraint. The voltage profile and losses of the distribution system is determined using NR load flow method from MATPOWER toolbox [43]. The available MATLAB programs for PFA [44] are modified to evaluate the proposed methodology and executed in a PC with specification of 4.00 GB, 64-bit OS and Intel^®^ Core™ i5-5200 CPU @ 2.30 GHz processor. The maximum number of iterations and also number of population are taken as 50.

### IEEE 33-bus system

**Fig. 4:**
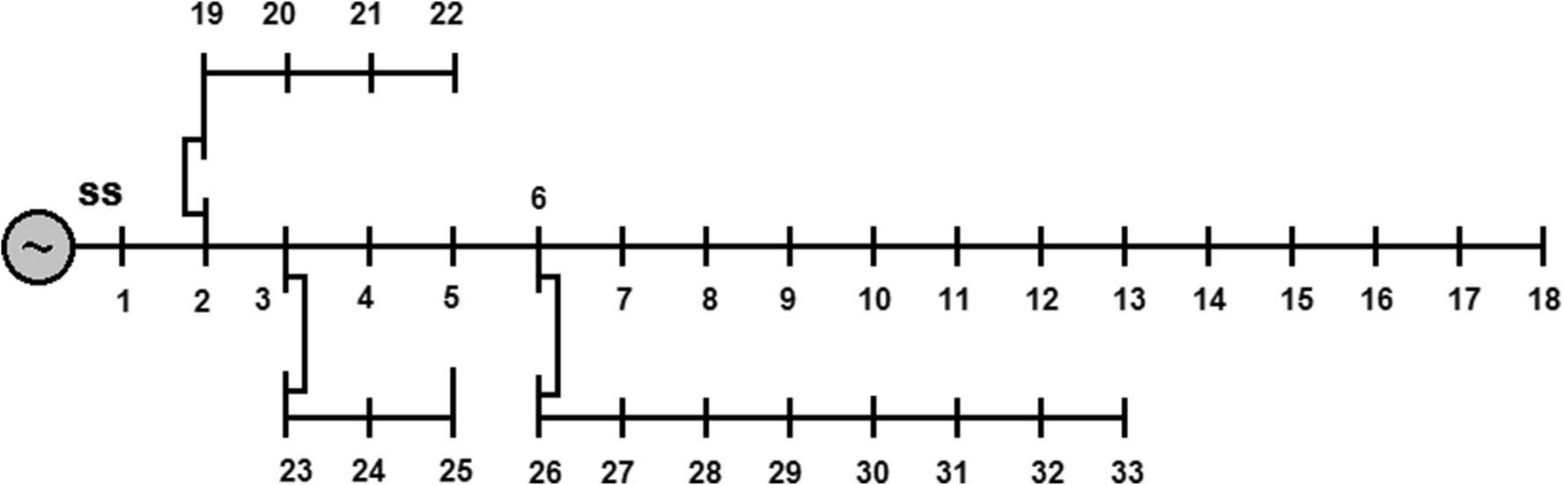
Single line diagram of standard IEEE 33-bus RDS

The single line diagram of IEEE 33-bus system is given in Fig. 4. In this system, it is assumed that the buses 1–18 as main feeder and buses 19–22, buses 23–25 and buses 26–33 as lateral feeders. From the data given in [41], the system is serving a load of (3715 kW + j 2300 kVAr) and suffering with total distribution losses of (210.9983 kW + j 143.0329 kVAr) respectively. Also, the system has poor voltage profile (i.e., < 0.95 p.u.) at some locations and the lowest voltage magnitude 0.9038 p.u is registered at 18th bus. The results of this base configuration are treated as Case-1.

#### Scenario-1: optimal location and sizing of PV system

Initially, PFA is applied to determine the optimal location and sizing of a single PV system considering loss minimization as objective, since the system has already satisfied radiality constraint. In order to simulate this scenario, the mathematical modeling explained in Sect. 2 is still applicable by neglecting interconnection branches. Also, the admittance of all branches are set to be zero, the DG location should be treated as load bus i.e., PQ bus and voltage magnitude should be considered as a control variable in search space. Considering these assumptions, this scenario is similar to many literature works solved for DG allocation with unity power factor (upf) in RDSs [16]. Hence, the search space for location and size are considered as [2, 33] and [0, 3715] respectively. The optimized solution by PFA is as follows: the best location: 6th bus; best PV size: 2590 kW; global optima: 111.0299 kW. Under these conditions, the feeder voltage profile is improved considerably with lower voltage magnitude 0.9424 p.u at 18th bus. The results of this stage are treated as Case-2.

**Fig. 5:**
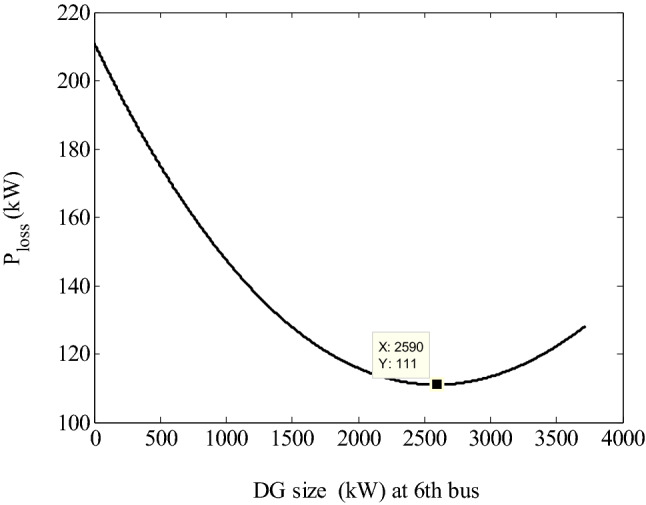
Impact of PV penetration at 6th bus on real power loss and optimal size

In order to verify the accuracy of the global solution provided by PFA, conventional NR load flow is repeated for incremental PV power step size as 1 kW up to 3715 kW. As it is shown in Fig. 5, the optimal PV size is 2590 kW at 6th bus and beyond this, again the losses increase. Hence, it can be said that the solution given by PFA is exactly a global minima.

As given in Table 1, the competitiveness of FPA with other HSAs is highlighted. It is observed that PFA is superior to WIPSO [8], SADE [8], ALO [20] and MFO [19] and well in agreement with the results of WOA [15], HGWO [6] and DA [16].

**Table 1:** Comparison of FPA with literature in IEEE 33-bus system

Method	PV (kW) and bus	P_loss_ (kW)	Q_loss_ (kVAr)	V_min_ (p.u.) and bus	AVDI	VSI and bus
Base	–	210.9983	143.0329	0.9038 (18)	0.0547	0.6486 (16)
WIPSO [8]	1600 (30)	125.267	89.590	0.9280 (18)	0.0328	0.7222 (16)
SADE [8]	1600 (30)	125.267	89.590	0.9280 (18)	0.0328	0.7222 (16)
ALO [20]	2450 (6)	111.302	81.702	0.9404 (18)	0.0294	0.7619 (16)
MFO [19]	2560 (6)	111.042	81.658	0.9419 (18)	0.0283	0.7670 (16)
WOA [15]	2589.6 (6)	111.030	81.683	0.9424 (18)	0.0280	0.7684 (16)
HGWO [6]	2590 (6)	111.030	81.684	0.9424 (18)	0.0280	0.7684 (16)
DA [16]	2590.2 (6)	111.030	81.684	0.9424 (18)	0.0280	0.7684 (16)
Proposed	2590.264 (6)	111.030	81.684	0.9424 (18)	0.0280	0.7684 (16)

**Fig. 6:**
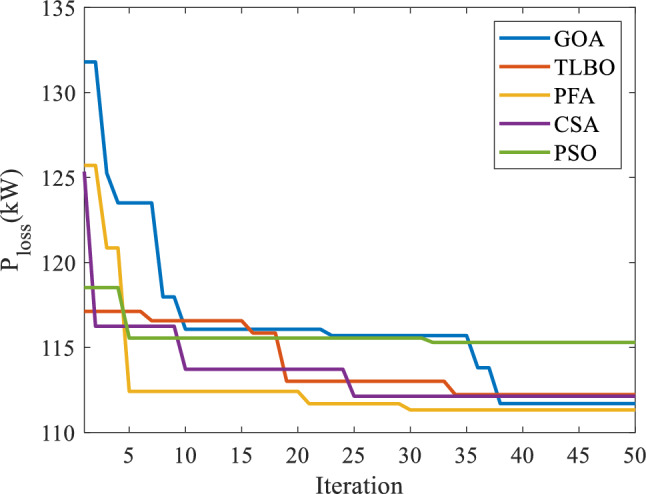
Convergence characteristics of PFA with other HSAs for Case-2 in IEEE 33-bus system

Table 2 explores the performance characteristics of proposed PFA and other heuristic algorithms namely particle swarm optimization (PSO) [45], teaching–learning based optimization (TLBO) [46], cuckoo search algorithm (CSA) [47], and grasshopper optimization algorithm (GOA) [48]. These techniques are implemented for solving Case-1. The results are highlighting the PFA superiority over other HSAs with lowest objective function value. In addition the best, mean and standard deviation (SD) values of PFA are also less than other HSAs considerably. The lowest best value (111.0299) indicates PFA as a global optimization algorithm, lowest mean value (111.2841) indicates its precision and lowest SD (0.0267) indicates its capability in avoiding local minima than other algorithms. As seen in the same table, the average computational time for 50 times independent trail runs is also less than other HSAs. At this stage, it can be concluded that the PFA is faster than other HSAs by 25.342 s. The convergence characteristics of PFA and other HSAs are given in Fig. 6.

**Table 2:** Comparison of PFA performance with other HSAs for 50 runs

Method	Performance measures			
	Best	Mean	SD	Time (s)
PSO	115.0025	111.7151	0.0295	31.021
TLBO	112.1362	110.8476	0.0291	29.002
CSA	112.1467	110.5194	0.0281	28.923
GOA	111.0302	111.2955	0.0275	25.453
Proposed PFA	111.0299	111.2841	0.0267	25.342

#### Scenario-2: optimal I-PV configuration with radiality constraint

**Fig. 7:**
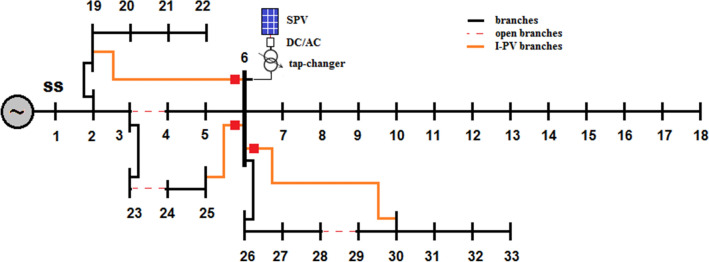
Optimal I-PV configuration in IEEE 33-bus system with 3 laterals

In order to form I-PV configuration, it is required to determine at least one bus on any lateral feeder for interconnecting the existing PV system on the main feeder. In this case, PFA needs to identify simultaneously for best location and size of PV system in the entire search space (i.e., [2, 33]) as well as for forming I-PV configuration with 3 laterals. It also needs to identify 3 interconnecting points among 15 buses (i.e., from bus-19 to bus-33) and 3 branches for opening among 22. Different case studies are performed. As given in Table 3, Case-3 is for 1 optimal lateral feeder, Case-4 is for 2 optimal lateral feeders, Case 5 is for 3 optimal feeders. In each case, the optimal size of PV system at 6th bus, the optimal interconnecting points on lateral feeders, optimally opened branches for radiality and correspondingly losses and lowest voltage profile are given in the same Table 3. Here, the results obtained for Case 5 are explained. Optimal PV location: 6th bus; PV size: 3356 kW; I-PV integration points on lateral 1 is 19th bus, lateral 2 is 25th bus and lateral 3 is 30th bus; and branch numbers to open: 3 (3–4), 23 (23–24) & 28 (28–29). The overall I-PV configuration with 3 laterals is given in Fig. 7. Under these conditions, the losses (51.7086 kW + j 54.8051 kVAr) and minimum voltage at 18th bus is 0.9469 p.u.

**Table 3:** Overall summery of all cases in IEEE 33-bus system

Case #	Bus numbers for I-PV configuration	Branch numbers for opening	PV (kW)	P_loss_ (kW)	Q_loss_ (kVAr)	V_min_ (p.u.) and bus	AVDI	VSI and bus	Computation time (s)
1	–	–	–	210.9983	143.0329	0.9038 (18)	0.0547	0.6486 (16)	0.283
2	6	–	2590	111.0299	81.6838	0.9424 (18)	0.0280	0.7684 (16)	0.442
3	6, 19	3	2685	81.2494	69.4196	0.9487 (18)	0.0242	0.7885 (16)	0.515
4	6, 19, 25	3, 23	3405	75.4461	70.5234	0.9469 (18)	0.0247	0.7836 (16)	0.553
5	6, 19, 25, 30	3, 23, 28	3356	51.7086	54.8051	0.9469 (18)	0.0209	0.7835 (16)	0.598
6	6, 19, 25, 30	–	3228	46.6756	44.1169	0.9508 (18)	0.0177	0.7967 (16)	0.601

#### Scenario-3: optimal I-PV configuration without radiality constraint

In Case 6, optimal I-PV configuration is determined with three laterals without considering radiality constraint. The optimized results of PFA are as follows: PV location: 6th bus; PV size: 3228 kW; I-PV integration points on lateral 1 is 19th bus, lateral 2 is 25th bus and lateral 3 is 30th bus. Under these conditions, the losses (46.6756 kW + j 44.1169 kVAr) and minimum voltage at 18th bus is 0.9508 p.u. There is almost 77.87% reduction in loss as compared to base case.

The impact of optimal I-PV configuration is evaluated in terms of average voltage deviation w.r.t. substation bus and voltage stability. The results of AVDI and VSI for each case are also given in the Table 3 In comparison, the AVDI is reduced as the number of interconnections increases on different lateral feeders. This indicates the improved voltage profile across the network with the I-PV system. Also, the increased VSI indicates the enhanced voltage stability margin in the system.

**Fig. 8:**
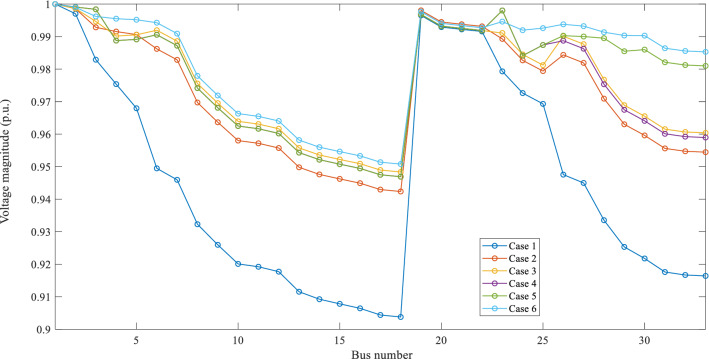
Voltage profile of IEEE 33-bus system under various case studies

The voltage profile under each case is given in Fig. 8. From the figure, it can be observed that the minimum voltage limit is satisfied only under Case 6 without maintaining radiality constraints.

### IEEE 69-bus system

**Fig. 9:**
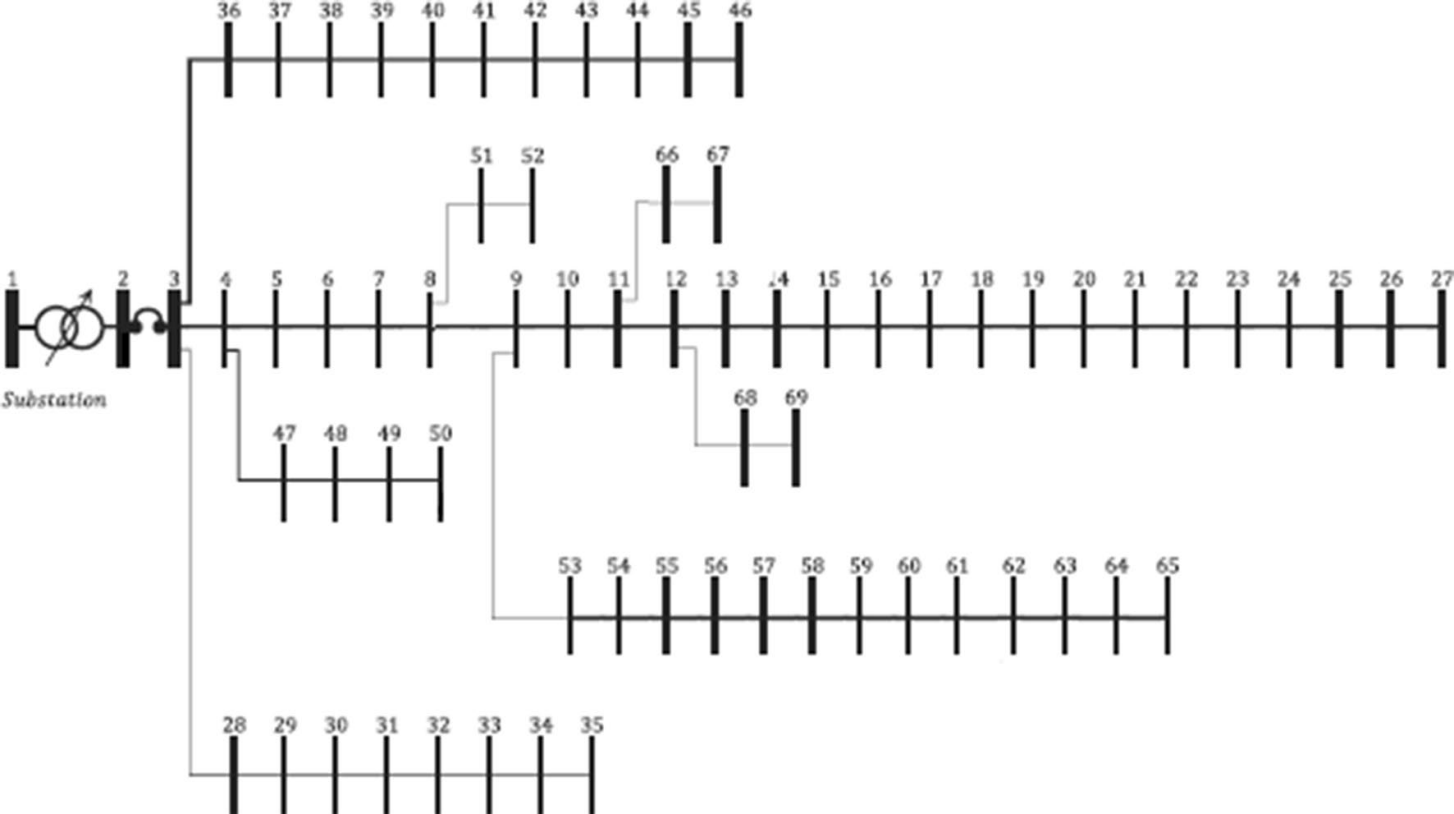
Single line diagram of IEEE 69-bus system

The single line diagram of IEEE 69-bus system is given in Fig. 9. It has 68 sectionalizing switches. In this system, it is assumed that the buses 1–27 as main feeder and 28–35, 36–46, 47–50, 51–52, 53–65, 66–67 and 68–69 as lateral feeders. The total load of the system is (3802.1 kW + j 2694.7 kVAr) and total loss is (225.0007 kW + j 102.1648 kVAr). The lowest voltage magnitude 0.9092 p.u. is registered at 65th bus. The results of this standard system are treated as Case-1.

#### Scenario-1: optimal location and sizing of PV system

By using the proposed PFA, the location and size of the PV type DG is optimized. The search space for location is [2, 69] and DG size is [0, 3802 kW] are considered. The proposed PFA determines the optimal size of the PV system is 1873 kW at bus-61. The best solution of loss is (83.224 kW + j 40.536 kVAr). The lowest voltage is observed at 0.9683 p.u on the 27th bus. In comparison to base case, the total losses are reduced by 63.01%. The results of this section are treated as Case-2.

The solution of PFA is compared with various other methods as given in Table 4. It has been observed that the FPA is better than GOA-CSA [21], ALO [13], SFLA [10] and DA [16]. From the comparison, it can be said that the PFA is a keen competitor to various HSAs and fine tunes the decision variables towards global optima irrespective of size of search space.

**Table 4:** Comparison of simulation results of PFA for single PV type DG with literature

Method	PV (kW) and bus	P_loss_ (kW)	Q_loss_ (kVAr)	V_min_ (p.u.) and bus	AVDI	VSI and bus
Base	–	225	102.165	0.9092 (65)	0.0266	0.55 (60)
GOA-CSA [21]	1990.712 (6)	203.895	90.502	0.9146 (65)	0.0235	0.5650 (60)
ALO [13]	1872.82 (61)	83.224	40.536	0.9683 (27)	0.0126	0.8583 (60)
SFLA [10]	1872.7 (61)	83.224	40.536	0.9683 (27)	0.0126	0.8583 (60)
DA [16]	1872.7 (61)	83.224	40.536	0.9683 (27)	0.0126	0.8583 (60)
Proposed	1872.7 (61)	83.224	40.536	0.9683 (27)	0.0126	0.8583 (60)

#### Scenario-2: optimal I-PV configuration with radiality constraint

**Fig. 10:**
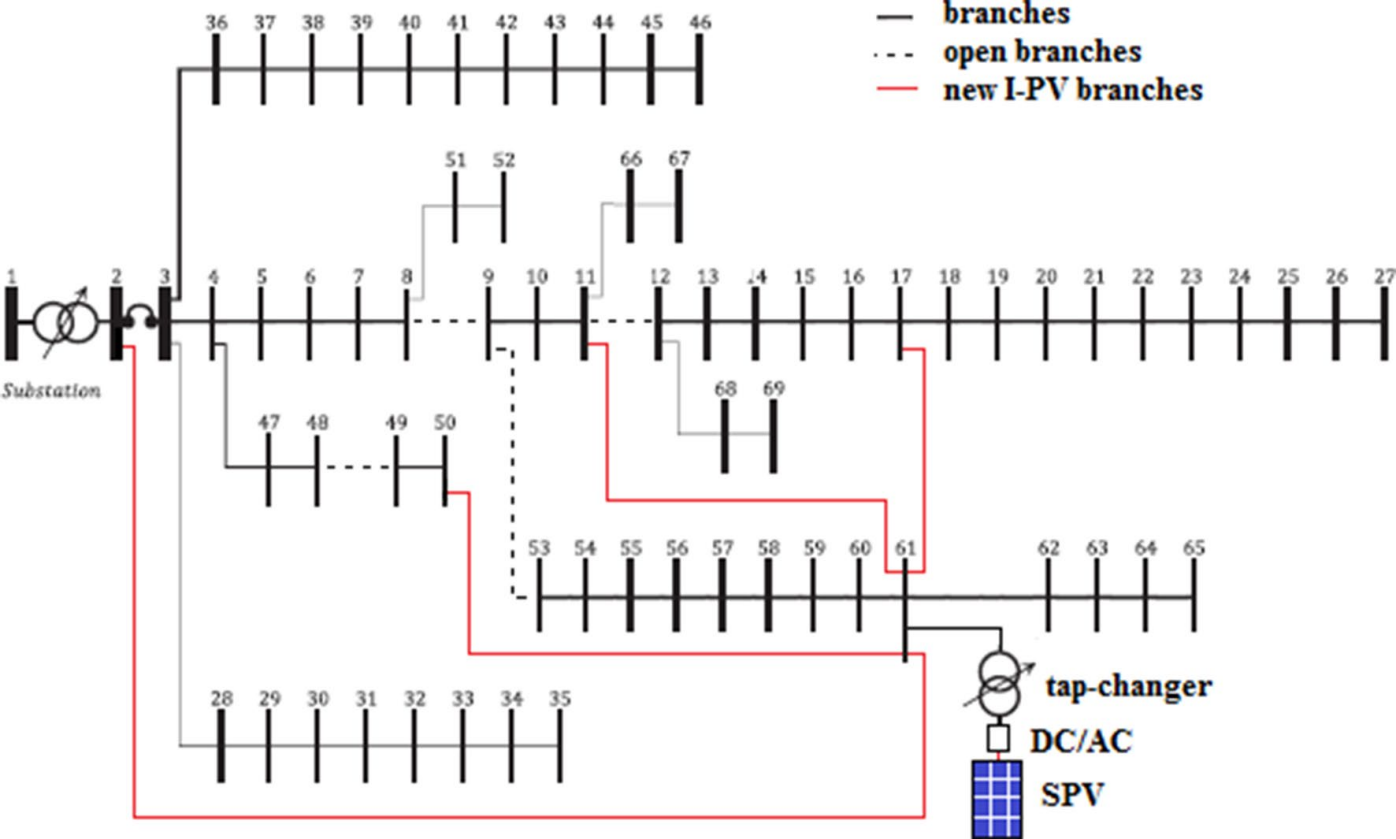
Optimal I-PV configuration in 69-bus system with 4 lateral feeders

As determined in IEEE 33-bus system, here also, it is required to determine at least one point on any lateral feeder for interconnecting the PV system. In this case, PFA needs to identify simultaneously the best PV location and size in the entire search space (i.e., [2, 69]) as well as best I-PV buses on lateral feeders. The new branch parameters of the I-PV system are chosen as the 68th branch in standard IEEE 69-bus system [42]. Different case studies are performed. As given in Table 5, Case-3 is for 1 optimal lateral feeder, Case-4 is for 2 optimal lateral feeders, Case-5 is for 3 feeders, and Case-6 is for 4 lateral feeders. In each case, the optimal size of PV system at 61st bus, the optimal integration points on lateral feeders, the optimal open branches for radiality and corresponding losses and lowest voltage profile is given in the same Table 4. Here, the results obtained for Case 6 are explained. Optimal PV location: 61st bus; PV size: 3340 kW; I-PV integration points on laterals are buses 2, 17, 11, 50 and the open branches: 52 (9–53), 11 (11–12), 8 (8–9) and 48 (48–49). Under these conditions, the losses (3.747 kW + j 1.875 kVAr) and minimum voltage at 69th bus is 0.9934 p.u. In comparison to the base case, the losses are reduced significantly by 98.33%, by which the network becomes almost independent from the grid. The single line diagram of 69-bus system with I-PV configuration using 4 lateral feeders is given in Fig. 10.

**Table 5:** Optimal I-PV configuration results in IEEE 69-bus

Case #	Bus numbers for I-PV configuration	Branch numbers for opening	PV (kW)	P_loss_ (kW)	Q_loss_ (kVAr)	V_min_ (p.u.) and bus	AVDI	VSI and bus	Computation time (s)
1	–	–		225	102.165	0.9092 (65)	0.0266	0.5500(60)	0.321
2	61	–	1872.7	83.224	40.536	0.9683 (27)	0.0126	0.8583(60)	0.535
3	61, 2	52	1924	23.814	14.907	0.9716 (27)	0.0085	0.8851(20)	0.572
4	61, 2, 17	52, 11	2429	7.406	8.111	0.9934 (69)	0.0018	0.9067(48)	0.593
5	61, 2, 17, 11	52, 11, 8	2645	5.914	7.371	0.9934 (69)	0.0014	0.9068(48)	0.625
6	61, 2, 17, 11, 50	52, 11, 8, 48	3340	3.747	1.875	0.9934 (69)	0.0012	0.9480(63)	0.659
7	61, 2, 17, 11, 50	–	3314	2.2159	1.3208	0.9971 (69)	0.0008	0.9110(48)	0.691

Here also, the impact of optimal I-PV configuration is evaluated in terms of average voltage deviation w.r.t. substation bus and voltage stability. The results of AVDI and VSI for each case are also given in Table 5. In comparison, the AVDI is reduced as the number of interconnecting points increases on different lateral feeders. This indicates the improved voltage profile across the network with the I-PV system. Also, the increased VSI indicates the enhanced voltage stability margin.

#### Scenario-3: optimal I-PV configuration without radiality constraint

**Fig. 11:**
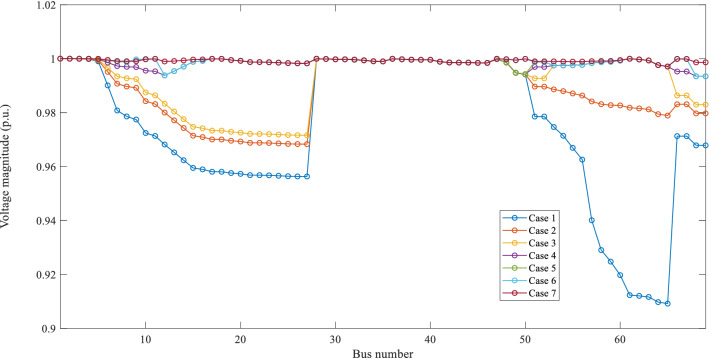
Voltage profile of IEEE 69-bus under different cases

The results of optimal I-PV configuration without radiality constraint are also given in Table 5. The optimized results of PFA are as follows: PV location: 61st bus; PV size: 3314 kW; I-PV integration points on laterals are the same as in Case-7. Under these conditions, the losses (2.2159 kW + j 1.3208 kVAr) and minimum voltage at 69th bus is 0.9971 p.u. The voltage profile under each case is given in Fig. 11. It can be observed that the voltage profile is almost flat throughout the feeder in Case-5 to Case-7 respectively. At this stage, it can be said that with more I-PV interconnections with lateral networks, the system performance can improve significantly in terms of reduced losses and increased voltage profile.

The impact of optimal I-PV configuration is evaluated in terms of average voltage deviation w.r.t. substation bus and voltage stability. The results of AVDI and VSI for each case are also given in Table 5. In comparison, the AVDI is reduced as the number of interconnecting points increases on different lateral feeders. This indicates the improved voltage profile across the network with the I-PV system. Also, the increased VSI indicates the enhanced voltage stability margin.

## Conclusion

In this paper, a new meta-heuristic Pathfinder Algorithm (PFA) is implemented for solving the simultaneous OADG problem and optimal I-PV configuration in RDSs. At first, the performance of the proposed PFA is evaluated first for solving OADG problem for loss minimization in IEEE 33-bus system and compared with PSO, TLBO, CSA and GOA by simulating 50 times each. The results are compared by analyzing best, mean, standard deviation of the fitness functions obtained in 50 runs and elapsed time. The comparison of these performance measures has clearly highlighted the superiority of PFA over other algorithms by providing global optima more often. Later, PFA is applied to determine optimal configuration of Interline-Photovoltaic (I-PV) system among multi-lateral feeders for improving the performance and resilience in distribution system operation and control without compromising the various operational and radiality constraints. The simulation results on IEEE 33- and 69-bus systems have shown the adaptability of proposed methodology for practical applications with reduced loss, improved voltage profile and enhanced voltage stability. Since, optimization of DGs’ location and sizes considering various operational and radiality constraints is a non-linear multi-objective optimization problem, application of PFA has resulted for global optima more often by avoiding the trap of local optima.

## Abbreviations

AVDI: Average voltage deviation index
DG: Distributed generation
EDS: Electrical distribution system
HSA: Heuristic search algorithm
IEEE: Institute of electrical and electronics engineers
I-PV: Interline-photovoltaic system
NR: Network reconfiguration
OADG: Optimal allocation of distribution generation
ONR: Optimal network reconfiguration
PFA: Pathfinder algorithm
RDS: Radial distribution system
RE: Renewable energy
VSI: Voltage stability index
